# Prevalence and characteristics of HIV drug resistance among antiretroviral treatment (ART) experienced adolescents and young adults living with HIV in Ndola, Zambia

**DOI:** 10.1371/journal.pone.0236156

**Published:** 2020-08-17

**Authors:** Sam Miti, Ray Handema, Lloyd Mulenga, Jonathan K. Mwansa, Elizabeth Abrams, Christiana Frimpong, Virginia M. Burke, Mangani Zulu, Mpanji Siwingwa, David Mwakazanga, Sam Kalibala, Julie A. Denison

**Affiliations:** 1 Arthur Davison Children’s Hospital, Ndola, Zambia; 2 Tropical Diseases Research Center, Ndola, Zambia; 3 University Teaching Hospital, Adult Infectious Diseases Center, Lusaka, Zambia; 4 Vanderbilt University Medical Center, Division of Infectious Diseases, Nashville, Tennessee, Zambia; 5 University of Zambia, School of Medicine, Division of Infectious Diseases, Lusaka, Zambia; 6 John Hopkins University Bloomberg School of Public Health, Department of International Health, Baltimore, Maryland, United States of America; 7 Population Council, Washington, DC, United States of America; International AIDS Vaccine Initiative, UNITED STATES

## Abstract

**Background:**

HIV drug resistance (HIVDR) poses a threat to the HIV epidemic control in Zambia especially in sub-populations such as the 15–24 years where there is poor virological suppression. Understanding the prevalence and patterns of HIVDR in this population (15–24 years) will contribute to defining effective antiretroviral therapy (ART) regimens, improving clinical decision making, and supporting behavioral change interventions needed to achieve HIV epidemic control.

**Methods:**

A cross-sectional analysis of study enrollment data from the Project YES! Youth Engaging for Success randomized controlled trial was conducted. Participants were 15 to 24 years old, who knew their HIV status, and had been on ART for at least 6 months. All participants completed a survey and underwent viral load (VL) testing. Participants with viral failure (VL ≥1,000 copies/mL) underwent HIVDR testing which included analysis of mutations in the protease and reverse transcriptase genes.

**Results:**

A total of 99 out of 273 analyzed participants receiving ART had VL failure, of whom 77 had successful HIVDR amplification and analysis. Out of the 77, 75% (58) had at least one drug resistant mutation, among which 83% (48/58) required a drug change. Among the 58 with HIVDR mutations, the prevalence of at least one HIVDR mutation to nucleoside reverse transcriptase inhibitors (NRTIs), non-nucleoside reverse transcriptase inhibitors (NNRTIs) and protease inhibitors (PIs) were 81%, 65.5% and 1.7%. The mutation M184V which confers resistance to NRTI drugs of lamivudine (3TC) and emtricitabine (FTC) was the most common (81%) among NRTI associated mutations followed by K65R (34.5%) which is associated with both tenofovir disoproxil fumarate (TDF) and tenofovir alafenamide fumarate (TAF) resistance. Thymidine analogue mutations (TAMs) which confer resistance primarily to zidovudine (AZT), stavudine (d4T) and other NRTIs were observed at 32.8%. Common TAMs were K70RTQNE (32.8%), K219QE (22.4%), D67N (17.2%) and T215IT (15.5%). The most common NNRTI associated mutation was the K103N (65.5%) which confers resistance to both efavirenz (EFV) and nevirapine (NVP). There was a relatively high occurrence of other NNRTI mutations V106A (36.2%), as well as Y188C (36.2%) and Y181C (36.2%) which confer resistance to etravirine.

**Conclusions:**

There is a high prevalence of HIVDR including TAMs despite majority of these patients (90.48%) being on AZT or d4T sparing first line ART among the youth. Emergence of these mutations including the NNRTI associated mutations (Y181C and Y188C) may compromise future second- and third-line regimens in the absence of routine HIVDR testing. HIVDR monitoring at start of ART or at first-line failure can better inform clinical decision making and ART programing.

## Introduction

The introduction of universal access to antiretroviral therapy (ART) for chronic HIV care management has been one of the greatest achievements of the last two decades for sub-Saharan Africa (SSA) where the burden of HIV is highest [1]. In Zambia, the number of people accessing ART has exponentially increased from 51,764 in 2005 to 1,076,000 by end of 2019 [2]. Unfortunately, corresponding with this increase in ART coverage are data from SSA that reveal a potential increase of HIV drug resistance (HIVDR) [3–6]. Emerging HIVDR data from Zambia are typically from research conducted among adults or among women and infants in prevention of mother to child transmission (PMTCT) studies. For instance, Handema et al found much HIV drug polymorphism but no HIVDR among 28 ART naïve **Zambian** adults prior to widespread use of ART in Zambia [7]. Subsequent studies showed increasing prevalence of HIVDR with one study showing HIVDR at 5.7% among 548 ART-naïve adults and 16% of 25 ART-experienced adults (including PMTCT exposure) in 2007–2008 [8]. Another study found that 98% of 66 **Zambian** adults failing first line ART had HIVDR in 2009–2012 [9]. Poppe et al. demonstrated an increasing prevalence of HIVDR in infants from 21.5 in 2007/2009 to 40.2% in 2014 and was mainly driven by the PMTCT practices [10]. Another study from Lusaka, Zambia showed the prevalence of NNRTI DRM prevalence was high (at or near 100%) in all first line therapy in HIV infected adult patients (age >15 years) attending the University Teaching Hospital Infectious Diseases Centre Advanced HIV Clinic [9].

While HIVDR is recognized as a serious threat to attaining the 90-90-90 goals [3, 4], there is paucity in data about the extent to which adolescents and young adults (AYA) are affected in sub-Saharan Africa. In Tanzania, 90% of children and adolescents less than 18 years with virologic failure had drug resistance mutations with 79% having multi-class drug resistance [11]. In Johannesburg South Africa, 56.8% of 230 PMTCT-exposed but newly diagnosed children under two years had Non-Nucleoside Reverse Transcriptase Inhibitor (NNRTI) HIVDR. In Zambia, a study of HIVDR in perinatally infected children showed an increasing and worsening pattern of HIVDR from 21% in 2007/9 to 40% in 2014 [10].While these studies contribute to our understanding of drug resistance, only the study in Tanzania included adolescents [11].

This paucity of adolescent data is a gap in our knowledge. Pediatric HIV treatment has led to an increased survival of perinatally infected children and adolescents creating a new generation of heavily treatment experienced adolescents and young adults [1, 12, 13]. It is well-established that adolescents, who are, by definition, in a critical development stage, often have greater challenges dealing with chronic illnesses and medications than adults [14–18]. Additionally, adherence to ART by adolescents tends to be lower than that of children or adults [1, 16, 19, 20] increasing the risk of adolescents having resistant strains of HIV and/or subsequently transmitting such drug resistant HIV strains.

The 2019 Zambia Population-based HIV Impact Assessment (ZAMPHIA) report highlights these risks with youth achieving the lowest viral suppression rates, 34% in females and 35.7% in males aged 15–24 years compared to 73.0% and 74% in male and female adults 45–59 years [21]. Preliminary data from targeted VL testing conducted at Arthur Davison Children’s Hospital in Ndola Zambia in 2016 found that about 50% of adolescents had viral load (VL) failure (defined as confirmed VL of ≥1,000 copies/mL) (unpublished data) [22]. The actual prevalence of HIVDR among adolescents and young adults in Zambia, however, is unknown. It is further not known the proportion of failure of viral suppression attributable to drug resistance versus incomplete adherence. Knowledge of resistance can also improve our ability to assess the effectiveness of empiric first- and second-line ART regimens for HIV care.

We thus analyzed baseline study enrollment data collected through a randomized controlled trial entitled “Project YES! Youth Engaging for Success” in order to determine the prevalence and characteristics of HIV drug resistance among ART- experienced youth, aged 15 to 24 years, attending four HIV care clinics in Ndola, Zambia.

## Methods

The cross-sectional analysis was conducted using study enrollment data from Project YES! a randomized controlled trial that tested a clinic-based peer mentoring program for HIV-positive adolescents and young adults (AYA) in four clinics in Ndola, Zambia. Project YES! study methods are described in detail elsewhere (ClinicalTrials.gov NCT04115813) [23]. In brief, key study eligibility criteria included being between 15–24 years of age, on ART for at least 6 months, aware of one’s HIV-positive status, and willing to participate in the study. AYA were consecutively sampled in each study clinic. Once consented and enrolled into the study, Interviews and blood draws for viral load and HIVDR were done after which study participants were randomized to the intervention or delayed intervention arms. Medical chart data were extracted. Only viral load results with viral loads of ≥1000 copies/mL were further analyzed for HIVDR using the Stanford University HIV drug resistance mutations database. Any resistance mutations were considered in light of what ART regimen the participant was taking, and drug switches recommendations made to the attending clinician.

### HIV treatment context

The recommended first-line ART regimens in Zambia at the time of this study comprised of two nucleoside or nucleotide reverse transcriptase inhibitors (NRTIs/NtRTIs) along with a non-nucleoside reverse transcriptase inhibitor (NNRTI). The two NRTIs/NtRTIs were tenofovir disoproxil fumarate (TDF) or zidovudine (ZDV) or abacavir (ABC) plus either lamivudine (3TC) or emtricitabine (FTC) whereas the NNRTI was either efavirenz (EFV) or nevirapine (NVP). The second line regimens comprised of two NRTIs (TDF or ABC or ZDV plus 3TC or FTC) sequentially selected based on which NRTIs/NtRTIs were used in first line and a boosted protease inhibitor (PI), lopinavir-ritonavir (LPV-r) or atazanavir-ritonavir (ATV-r). Third line ART typically consisted of tailor-made regimen based on HIVDR results. The typical drugs that were available in public health facilities offering third line ART at the time of the study included the NNRTI etravarine (rilpivarine and doravarine were not yet available) and the NRTIs lamivudine and emtricitabine (for M184V mutations). PIs included lopinavir and atazanavir for patients who demonstrated sensitivity on HIVDR testing. However, in some cases the PI darunavir boosted by ritonavir was available as a third line option. Finally, raltegravir was the only integrase inhibitor available for use as a third line ART. Dolutagravir had not been introduced to the public health sector until late in the intervention phase of this study and the HIVDR testing did not test for integrase inhibitor gene regions.

There had been several ART policy changes that affected ART regimens before the study. These had been implemented as part of Ministry of Health directives to health facilities over time and included drug switches from stavudine to zidovudine, nevirapine to efavirenz and more recently tenofovir to abacavir based regimes for children and adolescents. Occasionally, the drug changes were driven by drug availability, but this was rather rare. Unfortunately, there has been inadequate documentation of reasons for drug changes in most study participants medical charts.

### VL and HIVDR testing

Blood samples for HIV viral load testing were obtained and collected in EDTA anticoagulated bottles. HIV viral load was determined using the Cobas Ampliprep/CobasTaqman 96 machine (Roche Systems, Germany). Blood samples with a viral load of >1000 copies/mL (virologic treatment failure) were further subjected to an HIV drug resistance test using an Applied Biosystems Genetic Analyzer model 3500XL (Hitachi, Japan). HIV-1 RNA was extracted from 140 milliliter of plasma using the Qiagene QiAmp viral RNA mini kit (QIAGEN, Germany). Genotyping encompassed protease and codons 1–230 of reverse-transcriptase genes. Sequences were assembled and manually edited using Sequencer version 4.5 software (Gene codes, Ann Arbor, MI). The outcomes of the HIV resistance tests were reported using the Stanford HIV drug resistance database at http://hivdb.stanford.edu/. HIVDR was defined as the presence of at least one drug resistance–associated mutation (DRM) according to the World Health Organizations (WHO) 2009 Surveillance Drug Resistance Mutation list, using the Stanford Calibrated Population Resistance analysis tool (version 4.1 beta, available at http://hivdb.stanford.edu/). A cross-sectional analysis of all HIVDR results at study enrollment was done to determine HIVDR prevalence and characteristics among adolescents and young adults with virologic treatment failure.

### Ethical approval

Ethics approval was obtained from the ERES Converge Research Ethics Committee and the Johns Hopkins University Institutional Review Board (JHU-IRB) for the parent Project YES! study. The Ministry of Health -Zambia, through the National Health Research Authority, approved the conduct of the study in the four sites.

### Study sample

**Fig 1:**
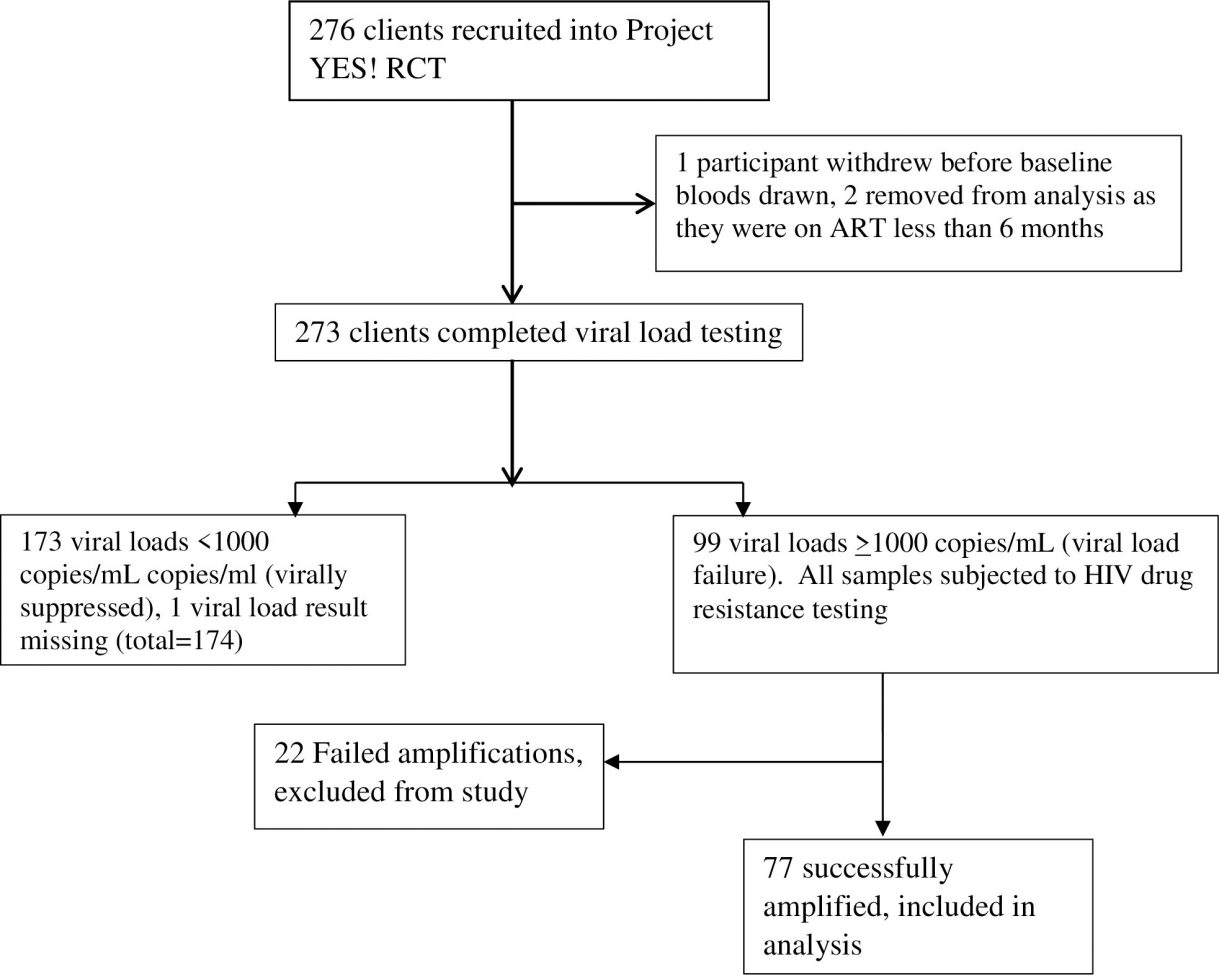
Flow chart of sampling process.

From December 2017 through May 2018, a total of 276 youth age 15 and 24 years from four clinics were recruited into the parent Project YES! study. One participant had missing study enrollment viral load results and two participants were removed from the analysis as data cleaning revealed they had been on ART for less than 6 months at start of the study. Among the 273 participants for whom study enrollment HIV-1 RNA viral load results were available, 99 participants (36.4%) had viral load ≥1000 copies/mL, signifying treatment failure. Out of these 99 participants, HIVDR amplification was successful in 77.8% (n = 77). A number of specimens with borderline viral loads failed to amplify. Fig 1 summarizes the process that resulted in the 77 participants included in this analysis.

### Data management and statistical analysis

Resistance results were originally entered into a Microsoft® Excel 2010 (Redmond, WA, USA) database and imported into SAS 9.4 (SAS Institute Inc., Cary, NC, USA) for further data cleaning, recoding and statistical analyses. The data included both continuous and categorical variables. Continuous variables, such as age, were described using means and standard deviations, and medians with value ranges and interquartile ranges (IQR’s). Categorical variables, such as presence of a particular mutation, were described using frequency and percentage distributions.

## Results

Among the 77 youth with an HIVDR test result, 51.7% were female, 48.3% were aged 3–10 years and 52% were aged 11–24 years at the start of ART, the mean age at the time of the study was 18.4 years, and 72% self-reported to have been perinatally infected (Table 1). Based on the medical record data, 95% of these participants had been on ART for an average of 11.5 years. Additional information of characteristics for the entire study population that had viral load test and HIVDR testing done is provided in a supplementary table (S1 Table: Characteristics of study participants by viral status and HIVDR amplification).

**Table 1:** Participants characteristics with successful HIVDR results (n = 77).

	n (%)
**ART Resistance**	
**Yes**	58 (75.3)
**No**	19 (24.7)
**Among Participants with Resistance**	n = 58
**Age at ART initiation**	
3–10 years	28 (48.3)
11–24 years	30 (51.7)
**Mean age at study enrollment in years (range)**	18.4 (15–23)
**Sex**	
Male	28 (48.3)
Female	30 (51.7)
**Health Facility Type**	
Pediatric Setting	33 (56.9)
Adult Setting	25 (43.1)
**Duration of Treatment**	
≤5 years	19 (32.8)
> 5 years	39 (67.2)
**Drug Regimen at baseline enrollment**	
**First line**	49 (84.5)
Tenofovir/Lamivudine/Efavirenz	44 (75.8)
Alternate first line regimens*	5 (8.6)
**Second line**	9 (15.5)
Zidovudine/Lamivudine/Lopinavir/ritonavir	3 (5.2)
Other second line regimens**	6 (10.3)
**Mode of Acquisition (self-reported)**	
Parental	43 (74.1)
Through sex	2 (3.5)
Other	3 (5.2)
Don’t know	10 (17.2)

*Alternate first line ART regimens were: Tenofovir/lamivudine/nevirapine (n = 2), Zidovudine/lamivudine/nevirapine (n = 1) and Abacavir/lamivudine/efavirenz (n = 2).

**Alternate second line ART regimens were: Tenofovir/lamivudine/atazanavir boosted by ritonavir (n = 1), Tenofovir/lamivudine/ lopinavir boosted by ritonavir (n = 1), Tenofovir/Zidovudine/lamivudine/lopinavir boosted by ritonavir (n = 1), Zidovudine/lamivudine/ atazanavir boosted by ritonavir (n = 1) and Abacavir/ lamivudine/ atazanavir boosted by ritonavir (n = 2).

HIV Drug Resistance associated Mutations (DRM) were identified in 75.3% (58/77) of all successful sequences. Of the 77 youth with HIVDR results, 64 were on first line and 13 were on second line ART (Table 2). The prevalence of any resistance was 76.6% (49/64) among youth on first line HIV treatment and 69.2% (9/13) among youth on second line HIV treatment.

**Table 2:** Resistance and drug change by first or second line of treatment.

	First Line (n = 64)	Second Line (n = 13)	All with HIVDR results (n = 77)
Any resistance	49	9	58
No resistance at all	15	4	19
Drug change needed	45	3	48
Drug change not needed	19	10	29

Among the 58 patients with any resistance 48 (83%) had resistance to a drug they were currently taking and needed to switch drugs to an effective ART regimen (Table 2). Out of the 48, 3 participants were on second-line treatment and needed to switch to third line, and 45 of the participants were on first line and needed to be changed to a second line ART regimen. 70% (45/64) of patients failing first line ART needed drug change (Table 2).

Of the 45 who needed to switch to second line, 42 where on tenofovir based regimen, 2 on abacavir and 1 on zidovudine based regimens. Of these (n = 45), 19 had the K65R mutation that confers resistance to tenofovir and abacavir but potentiates the effectiveness of zidovudine as a second line option. Additionally, 13.3% (6 out of the 45) needing first line drug change had the type 2 TAMs combination of T215Y, D67N and K70RTQNE which in combination diminish the effectiveness of zidovudine.

Drug mutations were categorized based on the WHO2017 drug mutation list and the Stanford HIV drug resistance database (Table 3). The most common resistance was to the NRTI group of drugs accounting for 81% (47/58) followed by NNRTI 65% (38/58). One patient had a major PI mutation (1.7%) and three had total PI-Accessory mutations (5.2%). Sixty-nine percent of clients had both NRTI and NNRTI mutations.

**Table 3:** Distribution of HIV drug resistance mutations in the sample with any HIVDR (n = 58).

Drug group	Mutation	Frequency of mutation, n (%)
**NRTI Mutations (n = 47)**	M184V	47 (81.0)
	K65KR	20 (34.5)
	K70RTQNE	19 (32.8)
	K219EQR	13 (22.4)
	D67N	10 (17.2)
	T215IT	9 (15.5)
	Y115F	8 (13.8)
	K223R	8 (13.8)
	V75M	7 (12.1)
	A62V	5 (8.62)
	L74IV	5 (8.62)
	M41L	3 (5.17)
	F77L	2 (3.44)
	D218E	2 (3.44)
	E203AK	2 (3.44)
	I94L	2 (3.44)
	F116Y	1 (1.72)
	Q151M	1 (1.72)
	K223E	1 (1.72)
	L109I	1 (1.72)
	L234I	1 (1.72)
	T139K	1 (1.72)
**NNRTI Mutations (n = 38)**	K103N	38 (65.5)
	V106A	21 (36.2)
	Y188CL	21 (36.2)
	Y181CV	21 (36.2)
	G190ASV	18 (31.0)
	K101EHPN	18 (31.0)
	E138AGQ	17 (29.3)
	A98G	13 (22.4)
	P225H	12 (20.7)
	V108I	10 (17.2)
	H221Y	8 (13.8)
	V179DEILT	8 (13.8)
	F227L	5 (8.60)
	L100I	5 (8.60)
	M230L	1 (1.72)
**PI-MAJOR Mutations (n = 1)**	N88S	1 (1.72)
**PI-ACCESSORY Mutations (n = 5)**	L10LF	1 (1.72)
	Q58E	3 (5.17)
	K20T	1 (1.72)

Overall, the 10 most common mutations were M184V (81%), K103N (65.5%), Y188C (36.2%), Y181C (36.2%), V106A (36.2), K65R (34.5%), K70RTQNE (32.8%), G190ASV (31.0%), K101EHP (31.0%) and E138AGQ (29.3%). The prevalence of mutations Y188C and Y181C was at 36.2% for and these (individually or in concert) confer resistance to the NNRTI drug etravirine. Among the NRTI mutations, the most common Thymidine Analogue Mutations (TAM’s) were K70RTQNE (32.8%), K219QE (22.4%), D67N (17.2%), T215IT (15.5%) and M41L (5.2%). The ten most common mutations to the drug class NNRTI included K103N (65.5%), V106A (36.2%), Y188C/L (36.2%), Y181C/V(36.2%), G190ASV(31%), K101EHP(31%), E138AGQ(29.3%), A98G(22.4%), P225H(20.7%), and V108I(17.2%). The N88S was the only major PI mutation (1.7%) and there were three PI-Accessory mutations of L10LF (1.7%), K20T (1.7%) and Q58E (5.2%). Additional information of mutations by ART regimen is contained in S2 Table (see S2 Table: Number of participants by First and Second line ART regimens with specific HIVDR mutations).

**Fig 2:**
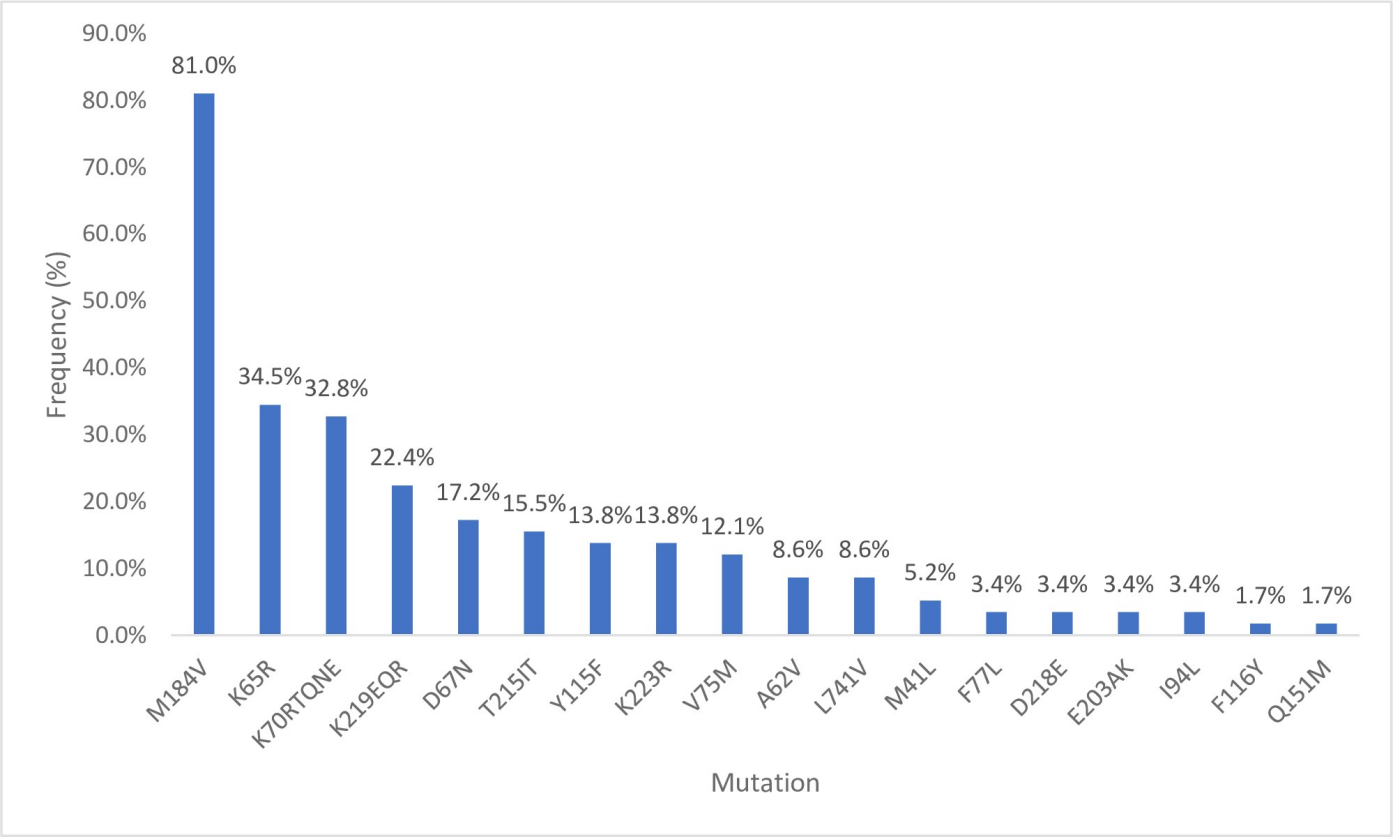
Frequency of individual NRTI mutations among patient with any HIVDR (n = 58).

**Fig 3:**
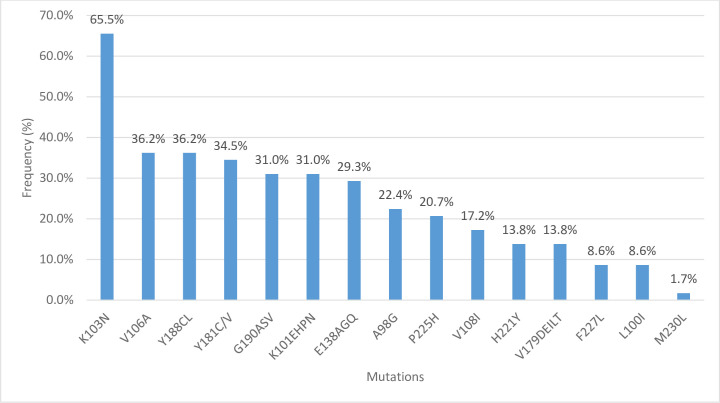
Frequency of individual NNRTI mutations among patient with any HIVDR (n = 58).

**Fig 4:**
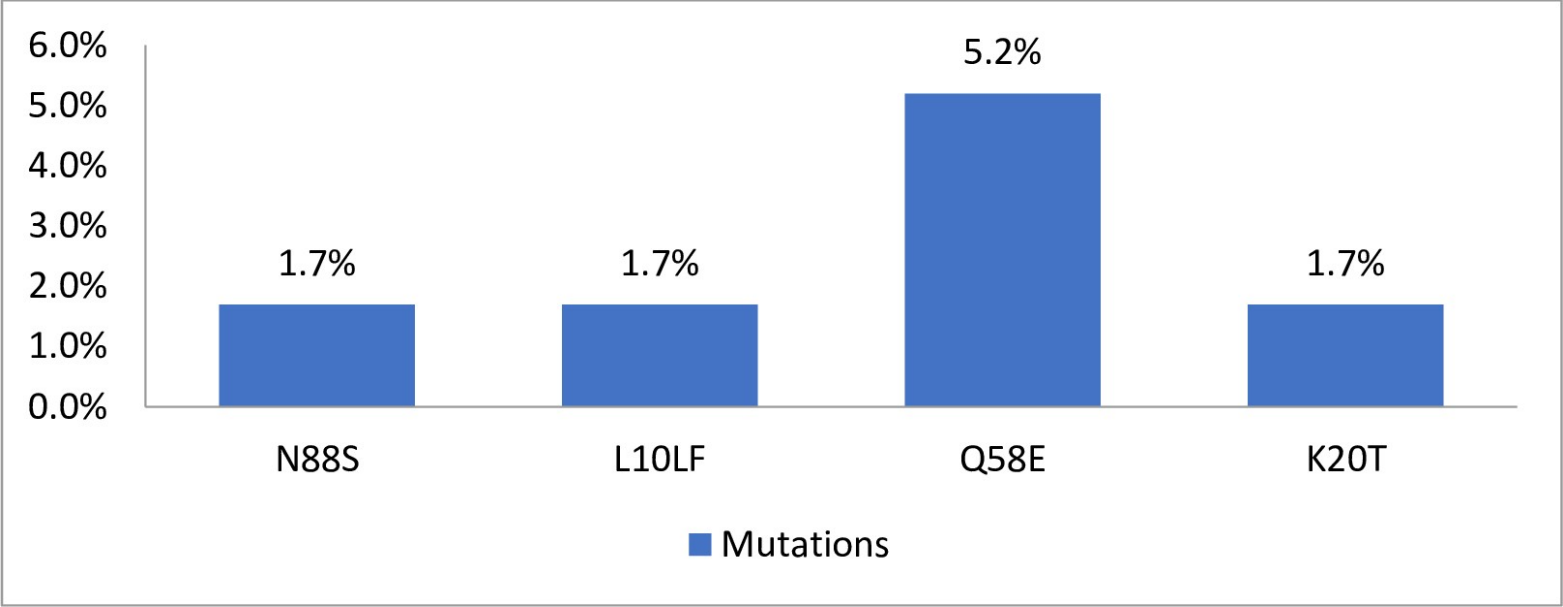
Frequency of individual PI mutations among patient with any HIVDR (n = 58).

Figs 2, 3 and 4 shows the frequency of clinically significant NRTI, NNRTI and PI mutations, respectively.

## Discussion

Our study provides critical data on resistance among adolescents and young adults with viral failure and attending clinical care in Ndola, Zambia and it has three key findings. The study population had similar characteristics for study participants who had successful HIVDR and those who did not. First, we observed a high level of HIV drug resistance, with three quarters (75.3%) of participants with viral load failure having at least one HIVDR mutation, and more than half (62%) showing drug resistance to at least one of their current ARV drugs. This suggests that almost two-thirds of youth with VL failure need an ART drug change while over a third may be failing due to reasons other than HIVDR, such as incomplete adherence and sub therapeutic dosing. It is also possible that participants with incomplete adherence could have HIVDR but with reversion to wild type. The phenomenon of reversion HIV to wild type in the absence of sufficient drug pressure may complicate the interpretation of HIVDR results for study participants who had virologic failure but no HIVDR. Reversion to HIV wild type has been documented in various settings and has been acknowledged as a challenge to interpretation of HIVDR results [24, 25].

High levels of HIVDR mutations are not unique to AYA or Zambia. Several studies among adults from Zambia, South Africa, Togo and Tanzania, have shown a range of HIVDR from 84–98% [26, 27]. The only study among children and adolescents conducted in Tanzania, found 90% had drug resistance with 79% having multiclass resistance.

Further, our study showed that most participants who had HIVDR to a current regimen were failing first-line ART (70%, 45/64) and needed a drug change to a second line regimen. This data highlights the potential role of delayed clinical decision making in this population. Interpretation of patient laboratory results solely lies with the attending health care workers. Factors that may contribute to delayed decision making may include inaccessibility of routine viral load test, delays in the turn-around times for viral load results (health systems delay with sample transportation, sample processing and transfer of results), as well as un-timely interpretation of results that are in patient medical records. There is therefore urgent need to strengthen mechanisms to ensure timely availability of viral load results, improve tracking mechanisms for results, and clinical supervision of health care workers to support timely decision making.

Secondly, this study found the prevalence of HIVDR mutations to first line NNRTI and NRTI was common (81% and 65% respectively). Only one patient (1.7%) had major protease inhibitor (PI) resistance mutation. This high prevalence of HIVDR mutations to NNRTI supports the 2020 Zambian HIV guidelines on the use of drugs with high genetic barriers, such as dolutegravir (DTG) for all age groups and protease inhibitors (lopinavir-ritonavir) for specific populations such as children ineligible for DTG [28] for first line treatment in place of NNRTIs.

As our study sampled ART treatment experienced AYA, we do not know the prevalence of primary transmitted resistance. However, studies from Zambia and sub-Saharan region have consistently shown an increasing prevalence of primary NNRTI resistance among adults (18 years or more) and pregnant women [12, 16, 29]. This increase in primary NNRTI resistance combined with the high percentage of AYA who have HIVDR to NNRTIs in this study sample indicates a pressing need for resistance testing in order to determine the best drug options for AYA, especially among perinatally infected youth. Further, only the Tanzanian study showed that 85% of children who had drug resistance developed HIV drug resistance while on treatment and that 15% of children and adolescents had primary HIVDR. This distinction is important because it may indicate unique factors at play for children and adolescents leading to drug resistance [11]. The Tanzania data also supports the importance of routine HIVDR surveillance to determine prevalence of primary and acquired HIVDR in both ART-naïve and -experienced children and adolescents to guide therapeutic options as they grow into young adults. It is therefore imperative that policies and mechanisms that prevent sub-therapeutic dosing and reinforce adherence among children as they continue to grow into AYA are re-examined.

In terms of HIVDR to NRTI, the prevalence of any TAMs was at 32.8% and the most common TAM’s were K70R/T/Q/N/E (32.8%), K219Q/E (22.4%), D67N (17.2%), T215IT (15.5%) and M41L (5.2%). TAM’s confer resistance to zidovudine, stavudine and other NRTI. When compared to earlier studies from Zambia [9], our study shows a higher prevalence of TAM’s among patients failing first line HIV treatment and with resistance. According to the Zambia Consolidated HIV guidelines 2018 (which were in use at the time of the study), zidovudine existed as part of a preferred second line regimen for patients failing on tenofovir or abacavir based regimens. The high prevalence of individual TAMs (D67N and K70R) or TAMs in combination (M41L with T215Y) reduces susceptibility to Zidovudine (a key drug for most second line regimens). The TAMs of K70R/T/Q/N/E, K219Q/E, and D67N in combination effectively reduce the potency of zidovudine in a second line combination. In our study, 13.3% (6 out of 45) of tenofovir based first line would have been potentially switched to a zidovudine based second line option that would have been sub-optimal. However, the majority of tenofovir based first line switches to zidovudine based second line would have been effective. It therefore means that potentially, more than 85% of patients with HIVDR failing on tenofovir/abacavir based first line and are blindly switched to zidovudine/protease inhibitor based second line without HIVDR testing may potentially be on a potent second line ART regimen with at least partial or full preserved zidovudine function.

It is concerning to see the high prevalence of TAMs despite the majority of these patients (90.4%) having been on zidovudine or stavudine sparing first line ART at the time of recruitment into the study. Since these patients were on tenofovir or abacavir, there is possibility that the TAMs observed were due to transmitted resistance or to archived mutations from prior drug switches/exposures. Transmitted resistance and archived mutations have reported as possible causes of HIVDR [25, 30]. The high prevalence of HIVDR mutations to NRTI and NNRTI may indicate the impact of previous policy driven first line drug changes over time that were not supported by viral load or HIVDR tests results. Most drug changes due to policy changes occurred within first line regimens (Zambia Consolidated guidelines 2012, 2014, 2018 and 2020). The impact of these policy driven drug changes on HIVDR in patients who were not clinically or immunologically failing (but who could have had drug resistance or virologic failure) may impact the interpretation of our HIVDR resistance mutations and results. These may also explain mutations that are usually selected by drugs that some study participants may not have been on at the time of study recruitment. For example, Zambia changed a policy to switch from stavudine or zidovudine, lamivudine and nevirapine as a preferred first line regimen to tenofovir, lamivudine and efavirenz as a preferred first line regimen (Zambia consolidated HIV guidelines 2012, 2014, 2018). Patients who may have had sub-clinical failure on stavudine or zidovudine-based ART regimen may have had archived HIVDR mutations. This may explain the high prevalence of TAMs in our study population. However, the high prevalence of TAMs was reported in an earlier study of adults failing HIV treatment in Lusaka, Zambia and other studies in the region [6, 8, 9, 10, 24]. Non the less, the poor documentation of the reasons for drug change in patient medical records remains a major limitation to interpreting the presence/absence of HIVDR mutations including TAMs. The finding of a high prevalence of TAMs in our study brings to question the current practice of switching patients failing first line regimens to second line regimens without resistance testing. Without resistance testing, we do not know what proportion of patients failing the first line ART where failing because of sub-optimal regimens or poor adherence. There is therefore an urgent need to provide HIV drug resistance testing at first line ART failure.

The third major finding of this study was the high prevalence of the Y188CE (36.2%) and Y181C (36.2%) mutations, indicating resistance to third line therapy etravirine and rilpivirine, to which none of our study participants had ever been exposed to. As a result, even before the restricted availability of etravirine in Zambia, more than one out of three (36%) patients with HIVDR may potentially be resistant to the only salvage NNRTI available in the public health sector in Zambia. The high prevalence of the Y181C and Y188C mutations has been demonstrated in earlier studies from the region including Zambia [9, 26, 11]. In our study, resistance to these drugs may be due in part to cross resistance with nevirapine and efavirenz which are widely used in PMTCT or first line ART. Unfortunately, we could not determine if the mutations to etravirine were due to primary HIVDR transmission or developed as secondary HIVDR. However recent data from the DAWNING trial and other studies [31, 32] have demonstrated the increasing superior role of integrase inhibitors over NNRTIs and PIs even in the presence of TAMs. These findings may mean that NRTIs retain some effectiveness when used in combination with integrase inhibitor dolutegravir. Therefore, the push to roll out integrase inhibitor dolutegravir as part of first and second line HIV care in Zambia may be timely in the face of our evidence that third line ARV etravirine may potentially not be effective in more than a third of all youth failing on ART in Ndola.

## Limitations

This analysis was a cross-sectional analysis among treatment-experienced participants and could not determine primary HIVDR or causal factors leading to HIVDR among AYA living with HIV. Data was also not collected on the PMTCT exposure/prior nevirapine use among participants or their parents. Non the less, we believe that our data makes a significant contribution to the database on prevalence and characteristics of HIVDR among AYA which remains limited in literature.

## Conclusions

HIVDR represents a serious challenge for Zambia’s national and regional health care systems. Many AYA with viral load failure may be resistant to their current regimens and in need of regimen change. Closer monitoring of drug resistant mutations at the start of HIV treatment or at first line failure can better inform clinical decision making and HIV treatment regimen programing.

## Supporting information

S1 Table Characteristics of study participants by viral status and HIVDR amplification (n = 272). (DOCX)

S2 Table Number of participants by first- and second-line ART regimens with specific HIVDR mutations (n = 58). (DOCX)
