# Supplementary material for: Prevalence and characteristics of HIV drug resistance among antiretroviral treatment (ART) experienced adolescents and young adults living with HIV in Ndola, Zambia
Source: PLoS One. 2020 Aug 17;15(8):e0236156. doi: 10.1371/journal.pone.0236156 (PMC7430722; doi:10.1371/journal.pone.0236156)
Supplement: S1 Table — (DOCX) [file pone.0236156.s001.docx]

**S1 Table. Characteristics of study participants by viral status and HIVDR amplification (n=272).**

| **Participant characteristics** | **VL**>**1000 and HIVDR, n=58(%)** | **VL**>**1000 and no HIVDR, n=19 (%)** | **VL**>**1000, HIVDR done but failed amplification, n=22 (%)** | **VL<1000, no HIVDR done, n= 173 (%)** |
| --- | --- | --- | --- | --- |
| **Age at ART initiation** |  |  |  |  |
| 3-10 years | 28 (48.3) | 6 (31.6) | 6 (27.3) | 52 (30.1) |
| 11-24 years | 30 (51.7) | 13 (64.4) | 16 (72.7) | 121 (69.9) |
| **Mean age in years (range)** | 18.4 (15-24) | 18.7 (15-24) | 19.2(15-24) | 19.2(15-24) |
| **Sex** |  |  |  |  |
| Female | 30 (51.7) | 11 (57.9) | 14 (63.6) | 107(61.8) |
| Male | 28 (48.3) | 8 (42.1) | 8 (36.4) | 66 (38.2) |
| **Health Facility Type** |  |  |  |  |
| Pediatric Setting | 33 (56.9) | 15 (78.9) | 12 (54.5) | 61 (35.3) |
| Adult Setting | 25 (43.1) | 4 (21.1) | 10 (45.5) | 112 (64.7) |
| **Duration of Treatment** |  |  |  |  |
| <5 years | 19 (32.8) | 6 (31.6) | 8 (36.4) | 69 (39.9) |
| > 5 years | 39 (67.2) | 13 (68.4) | 14 (63.6) | 145 (60.1) |
| **Drug Regimen at enrollment**  **First line** |  |  |  |  |
| Preferred 1st line-TDF/3TC/EFV | 44 (75.9) | 14 (73.7) | 16 (72.7) | 131 (75.7) |
| Alternate 1st line regimens*  **Second line** | 5 (8.6) | 0 (0) | 1 (4.5) | 9 (5.2) |
| Preferred 2nd line-AZT/3TC/LPV/r | 3 (5.2) | 3 (15.8) | 1 (4.5) | 17 (9.8) |
| Other second line regimens** | 6 (10.3) | 2 (10.5) | 4 (18.2) | 16 (9.2) |
| **Mode of Acquisition (self-reported)** |  |  |  |  |
| Parental | 43 (74.1) | 15 (78.9) | 16 (72.7) | 124 (71.7) |
| Through sex | 2 (3.5) | 1 (5.3) | 2 (9.1) | 22 (12.7) |
| Other | 3 (5.2) | 2 (10.5) | 1 (4.5) | 8 (4.6) |
| Don’t know | 10 (17.2) | 0 | 3 (13.6) | 18 (10.4) |
| Refused to answer | 0 | 1 (5.3) | 0 | 1 (0.6) |

***Alternate first line ART regimens were:** Tenofovir/lamivudine/nevirapine, Zidovudine/lamivudine/nevirapine, and abacavir/lamivudine and efavirenz.

****Alternate second line ART regimens were:** Tenofovir/lamivudine/atazanavir boosted by ritonavir **,** Tenofovir/lamivudine/ lopinavir boosted by ritonavir, Tenofovir/Zidovudine/lamivudine/lopinavir boosted by ritonavir , Zidovudine/lamivudine/ atazanavir boosted by ritonavir and Abacavir/ lamivudine/ atazanavir boosted by ritonavir.
