# Supplementary material for: Prevalence and characteristics of HIV drug resistance among antiretroviral treatment (ART) experienced adolescents and young adults living with HIV in Ndola, Zambia
Source: PLoS One. 2020 Aug 17;15(8):e0236156. doi: 10.1371/journal.pone.0236156 (PMC7430722; doi:10.1371/journal.pone.0236156)
Supplement: S2 Table — (DOCX) [file pone.0236156.s002.docx]

**S2 Table. Number of participants by first- and second-line ART regimens with specific HIVDR mutations (n=58).**

|  | First Line Regimen (n= 49) | | | | Second Line Regimen (n=9) | | | |  |  |
| --- | --- | --- | --- | --- | --- | --- | --- | --- | --- | --- |
| HIVDR  Mutation | **TDF/**  **3TC/**  **EFV** | **TDF/**  **3TC/**  **NVP** | **ABC/**  **3TC/**  **EFV** | **AZT/**  **3TC/**  **NVP** | **TDF/**  **3TC/**  **ATV/r** | **TDF/**  **3TC/**  **LPV/r** | **TDF/**  **AZT/ 3TC/LPV/r** | **AZT/**  **3TC/**  **LPV/r** | **AZT/**  **3TC/**  **ATV/r** | **ABC/**  **3TC/**  **ATV/r** |
| NRTI (n=47) |  |  |  |  |  |  |  |  |  |  |
| M184V (47)  K65KR (20)  K70RTQNE (19  K219EQR(13)  D67N (10)  T215IT (9)  Y115F (8)  K223R (8)  V75M (7)  A62V (5)  L74IV (5)  M41L (3)  F77L (2)  D218E (2)  E203AK (2)  I94L (2)  F116Y (1)  Q151M (1) | 37  18  17  10  8  7  6  7  5  5  4  2  2  1  2  1  1  1 | 2  1  0  1  0  0  1  0  1  0  0  0  0  0  0  1  0  0 | 1  0  0  0  0  1  0  0  0  0  0  1  0  0  0  0  0  0 | 1  0  0  0  0  0  0  0  0  0  0  0  0  0  0  0  0  0 | 1  0  0  1  1  1  0  1  0  0  0  0  0  0  0  0  0  0 | 0  0  0  0  0  0  0  0  0  0  0  0  0  0  0  0  0  0 | 0  0  0  0  0  0  0  0  0  0  0  0  0  0  0  0  0  0 | 3  1  1  1  0  0  1  0  1  0  1  0  0  1  0  0  0  0 | 0  0  0  0  0  0  0  0  0  0  0  0  0  0  0  0  0  0 | 2  0  1  0  1  0  0  0  0  0  0  0  0  0  0  0  0  0 |
| NNRTI |  |  |  |  |  |  |  |  |  |  |
| K103N (38)  V106A (21)  Y188CL (21)  Y181CV (21)  G190ASV (18)  K101EHPN (18)  E138AGQ (17)  A98G (13)  P225H (12)  V108I (10)  H221Y (8)  V179DEILT (8)  F227L (5)  L100I (5)  M230L (1) | 29  13  19  15  15  16  12  11  11  9  5  4  3  5  1 | 0  1  1  1  0  1  0  0  0  0  0  0  0  0  0 | 2  0  0  0  0  0  1  0  1  0  0  1  0  0  0 | 1  1  0  0  0  0  0  0  0  0  1  0  0  0  0 | 1  1  0  0  0  0  1  0  0  1  0  0  0  0  0 | 0  1  0  1  1  0  0  0  0  0  0  0  1  0  0 | 0  0  0  1  1  0  0  1  0  0  1  1  0  0  0 | 3  2  1  2  0  1  3  1  0  0  1  2  1  0  0 | 1  1  0  0  0  0  0  0  0  0  0  0  0  0  0 | 1  1  0  1  1  0  0  0  0  0  0  0  0  0  0 |
| PI |  |  |  |  |  |  |  |  |  |  |
| N88S (1)  L10LF (1)  Q58E (3)  K20T (1) | 0  0  0  0 | 0  0  0  0 | 0  0  0  0 | 0  0  0  0 | 0  0  0  0 | 0  0  0  0 | 0  1  0  0 | 1  0  2  1 | 0  0  0  0 | 0  0  1  0 |
|  |  |  |  |  |  |  |  |  |  |  |

Note:

1^st^ line ART regimens

TDF/3TC/EFV-Tenofovir/lamivudine/efavirenz

TDF/3TC/NVP- Tenofovir/lamivudine/nevirapine

ABC/3TC/EFV-Abacavir/lamivudine/nevirapine

AZT/3TC/NVP-Zidovudine/lamivudine/nevirapine

2^nd^ line ART regimens

TDF/3TC/ATV/r-Tenofovir/lamivudine/atazanavir boosted by ritonavir

TDF/3TC/LPV/r-Tenofovir/lamivudine/lopinavir boosted by ritonavir

TDF/AZT/3TC/LPV/r- Tenofovir/zidovudine/lamivudine/lopinavir boosted by ritonavir

AZT/3TC/LPV/r-Zidovudine/lamivudine/lopinavir boosted by ritonavir

AZT/3TC/ATV/r-Zidovudine/lamivudine/atazanavir boosted by ritonavir

ABC/3TC/ATV/r-Abacavir/lamivudine/atazanavir boosted by ritonavir

ABC/3TC/LPV/r-Abacavir/lamivudine/lopinavir boosted by ritonavir
